# The Role of Immunosenescence in Cerebral Small Vessel Disease: A Review

**DOI:** 10.3390/ijms23137136

**Published:** 2022-06-27

**Authors:** Alessandro Del Cuore, Gaetano Pacinella, Renata Riolo, Antonino Tuttolomondo

**Affiliations:** 1 Department of Promoting Health, Maternal-Infant, Excellence and Internal and Specialised Medicine (PROMISE) G. D’Alessandro, University of Palermo, 90133 Palermo, Italy; pacinella66@gmail.com (G.P.); renatariolo.rr@gmail.com (R.R.); bruno.tuttolomondo@unipa.it (A.T.); 2 Internal Medicine and Stroke Care Ward, Policlinico “P. Giaccone”, 90127 Palermo, Italy

**Keywords:** CSVD, ArCSVD, immunosenescence, inflammaging, BBB leakage, Endothelieal dysfunction

## Abstract

Cerebral small vessel disease (CSVD) is one of the most important causes of vascular dementia. Immunosenescence and inflammatory response, with the involvement of the cerebrovascular system, constitute the basis of this disease. Immunosenescence identifies a condition of deterioration of the immune organs and consequent dysregulation of the immune response caused by cellular senescence, which exposes older adults to a greater vulnerability. A low-grade chronic inflammation status also accompanies it without overt infections, an “inflammaging” condition. The correlation between immunosenescence and inflammaging is fundamental in understanding the pathogenesis of age-related CSVD (ArCSVD). The production of inflammatory mediators caused by inflammaging promotes cellular senescence and the decrease of the adaptive immune response. Vice versa, the depletion of the adaptive immune mechanisms favours the stimulation of the innate immune system and the production of inflammatory mediators leading to inflammaging. Furthermore, endothelial dysfunction, chronic inflammation promoted by senescent innate immune cells, oxidative stress and impairment of microglia functions constitute, therefore, the framework within which small vessel disease develops: it is a concatenation of molecular events that promotes the decline of the central nervous system and cognitive functions slowly and progressively. Because the causative molecular mechanisms have not yet been fully elucidated, the road of scientific research is stretched in this direction, seeking to discover other aberrant processes and ensure therapeutic tools able to enhance the life expectancy of people affected by ArCSVD. Although the concept of CSVD is broader, this manuscript focuses on describing the neurobiological basis and immune system alterations behind cerebral aging. Furthermore, the purpose of our work is to detect patients with CSVD at an early stage, through the evaluation of precocious MRI changes and serum markers of inflammation, to treat untimely risk factors that influence the burden and the worsening of the cerebral disease.

## 1. Introduction

**Figure 1:**
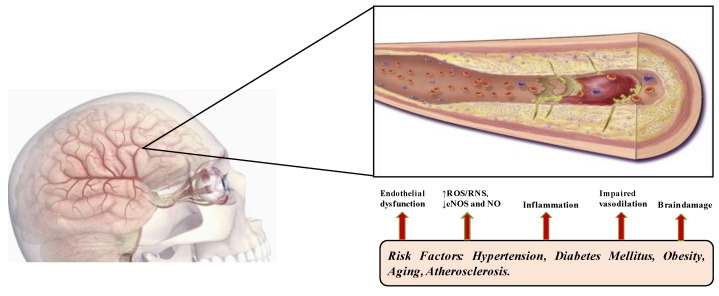
The pathophysiology of ArCSVD is complex and involves various players: the synergy of the altered molecular mechanisms that the various risk factors cause leads to tissue damage; diabetes mellitus, ageing, atherosclerosis, obesity and arterial hypertension cause an inflammatory microenvironment that influences endothelial dysfunction, promotes vasoconstriction by reducing the bioavailability of nitric oxide (NO), promotes the breakdown of the blood–brain barrier and the activation of aberrant immune mechanisms which, in an interminable vicious circle, amplify brain damage.

Deterioration of the central nervous system associated with age depends on different mechanisms related to chronic inflammation and the consequent decline in the immune response. With aging, the dysregulation of molecular mechanisms of the immune system promotes neurodegeneration and plays a crucial role in determining outcomes such as dementia and stroke. Cerebral small vessel disease (CSVD) is nowadays recognized as one of the most important causes of vascular dementia: immunosenescence and inflammatory response, with involvement of the brain’s vascular system, constitute the basis of this disease [1]. The definition of CSVD refers to all the pathological processes in which there is an involvement of small vessels of the brain: small arteries, capillaries, and small veins. This vascular disease is associated with the lacunar lesion, cortical atrophy, microbleeds, abnormal changes of white matter and expanded perivascular spaces. The etiopathogenic classification of cerebral small vessel disease identifies many types, but the most frequent forms are arteriolosclerosis-related, also known as age-related, and the cerebral amyloid angiopathy [2]. Age-related CSVD (ArCSVD) is the most important cause of brain infarct and vascular dementia. It strictly correlates to many risk factors such as aging, hypertension, smoking and diabetes [3,4,5] (Figure 1).

Although the concept of CSVD is broader, this manuscript focuses on describing the neurobiological basis and immune system alterations behind cerebral aging. Furthermore, the purpose of our work is to detect patients with CSVD at an early stage, through the evaluation of precocious MRI changes and serum markers of inflammation, in order to treat untimely risk factors that influence the burden and the worsening of the cerebral disease.

## 2. The Role of Aging in Pathophysiology of ArCSVD

Aging is characterized by immune-related modifications that determine susceptibility to infectious diseases, increased risk of cancer and cardiovascular diseases, and reduction in the effectiveness of vaccines. These conditions depend on a decline in the immune system called “immunosenescence” [6].

Immunosenescence identifies a condition of deterioration of the immune organs and consequent dysregulation of the immune response caused by cellular senescence, which exposes older adults to vulnerability [7].

A low-grade chronic inflammation status also accompanies it without overt infections, a condition named “inflammaging”, inflammation is a valid mechanism to destroy micro-organisms and harmful substances entering the human body, but chronic subclinical inflammation status associated with senescence increases the risk of degenerative and metabolic disease [8].

Under normal conditions, the innate and adaptive immune systems recognize dangerous substances and harmful stimuli such as pathogens [9] (bacteria, viruses, fungi, and parasites considered nonself), endogenous damaged cells, and degradation products of molecules (self) or gut microbiota (quasi-self), and activate the correct inflammatory response to maintain homeostasis. However, with aging, the degeneration of sensors that trigger the immune response causes uncontrolled activation of immune receptors and therefore of molecular cascades: pattern recognition receptors (PRRs) such as toll-like receptors (TLRs) 9 and NOD-like receptors lose their physiological functions and produce an aberrant inflammatory response that promotes chronic inflammation [9].

This mechanism produces cellular damage and accumulation of molecular debris in multiple tissues and organs of the human body, interfering with the repair processes [10].

Inflammaging is the final and long-term result of inexorable and incessant physiological stimulation of the immune system due to many cellular and molecular pathways, such as senescence of immune and non-immune cells, mitochondrial dysfunction, alteration of autophagy and metaflammation [11].

The recent scientific literature has shown that senescent cells (SCs) have adverse effects on tissue homeostasis by producing inflammation amplifiers. It seems that SCs promote inflammaging through a senescence-associated secretory phenotype (SASP), characterized by a broad series of inflammatory actors (interleukin-6, interleukin-8) and degradation products of extracellular matrix [12].

The presence of cellular debris and nondegradable products in the tissues triggers the innate immune response through the molecular cascade of “damage-associated molecular patterns” (DAMPs) that recognize self-signals and produce macrophage activation [13].

Innate immune activation promotes pro-inflammatory pathways through “nuclear factor kappa-light-chain enhancer of activated B cells” (NF-kB) and “signal transducer and activator of transcription” (STAT): the immune cells produce a significant number of inflammatory mediators (cytokines and chemokines) that amplify immune involvement [14].

In the pathophysiology of ArCSVD, the state of chronic inflammation caused by cellular aging and dysregulation of the immune system leads to endothelial dysfunction and alteration of the blood–brain barrier: it seems that this process could be identified early through the detection of circulating biological markers [15] categorized as systemic inflammatory factors such as CRP and IL-6 or vascular/endothelial altered factors such as homocysteine and von Willebrand factor.

Moreover, many studies have underlined that the early identification of pathological modifications of the BBB through imaging techniques such as classical MRI or functional MRI could predict the cognitive impairment in aged patients [16].

BBB leakage and endothelial dysfunction are considered the progenitors in the development of ArCSVD. Early correction of the risk factors underlying these two alterations is considered the best treatment to halt cognitive decline.

The BBB dysfunction leads to the release of the central nervous system (CNS) antigens into the peripheral circulation and the infiltration of leukocytes into brain tissue [17,18].

The transit of serum proteins at the neurovascular unit due to BBB dysfunction leads to microglia activation. Macrophages of microglia can produce chemokines, which provoke the migration of peripheral inflammatory cells to the CNS, generating a perpetual inflammatory microenvironment and supporting activated lymphocytes to meet CNS antigens [19,20,21,22,23,24].

Therefore, immunosenescence and the set of altered molecular mechanisms described above are the basis for understanding many diseases in older people: cellular aging damages the immune system both in its innate and adaptive compartments.

## 3. Innate and Adaptive Aging-Related Immune System Alterations

The innate immune response constitutes the first barrier of the human body against pathogens and harmful substances [25].

The cellular elements of this compartment can recognize conserved pathogen-associated molecular patterns (nonself) and autologous antigens of the damage-associated molecular patterns (self) through different specific types of sensors [26].

Beyond this, the revolutionary concept that has been increasingly understood in recent years is that the innate immune system has a kind of memory, named “innate immune memory”, which can recognize dangerous signals through a few receptors and amplify the response by using many effectors in a model known as “bow-tie” [9].

Furthermore, it was observed that after some time from a specific activation (Calmette-Guérin bacillus, BCG), innate immune cells were able to recognize the trace of the previous activation and to react in the absence of BCG to other stimuli through this “innate memory” [27].

The knowledge of these elements and complex mechanisms makes it possible to understand the complexity of the action of the innate immune system.

The aging processes of innate immunity are related to dichotomous mechanisms: immune stimulation and immune paralysis, exacerbation of some functions coexisting with depression of others. This peculiarity, in the past, was considered the hinge of inflammaging through the excessive production of inflammatory mediators by senescent immune cells and the consequent boost of adaptive immunity and amplification of low chronic inflammation status. Recent observations, however, have shown that inflammaging is the result of many alterations such as mitochondrial aging, resident microbiota changes, and oxidative stress (oxy-inflammaging) [28].

The trained innate immune memory described above could be, according to several studies, the promoter of immune activation in the absence of specific triggers. It is mainly constituted through epigenetic modifications and probably concerns the molecular pathways of energy production, thereby generating a significant change in the mechanisms of immunomodulation: macrophages, which are the protagonists of innate immunity, can modify their phenotype and the pattern of inflammatory molecules produced, playing a pivotal role in determining the state of low-grade chronic inflammation that characterizes the elderly, and it seems likely that many of these changes happen in the epigenomic field [7].

Chronic stresses determine intracellular modifications and deterioration of molecular mechanisms over the years—mitochondrial dysfunction, impaired autophagy, and damage to DNA repair processes. It implies that, with aging, the balance between pro-inflammatory and anti-inflammatory molecules is lost, and there is a preponderance of inflammation status: the consequence of this mismatch is the damage of the whole organism at different levels [29]. 

The counterbalance of chronic low-grade inflammation that represents the hyperactivation of the immune system in the elderly is immune paralysis. In physiological conditions, this mechanism protects against the activation of immune cells against autologous antigens and avoids self-damage. However, with aging, the functional alterations cause the loss of protective activity against pathogens and other harmful substances, reinforcing the condition of fragility that makes it more likely for an older adult to fall ill [30].

However, the condition of immune paralysis provides some advantages in the elderly organism because it reduces the energy expenditure of the innate immune system: the most explicit example is macrophages, for which it is too expensive to maintain the M1 phenotype (pro-inflammatory and antitumor). At the same time, it is much more energetically convenient to acquire the M2 phenotype (angiogenetic and cancer-growth-promoting) [31].

Therefore, the crucial task, for the aged organism, is to maintain a convenient balance between the need to fight pathogens and the pathway of chronic inflammation to avoid tissue damage. Mitochondria come into play in this challenging process. Some degree of mitochondrial dysfunction is responsible for successful aging as opposed to overall mitochondrial dysfunction, responsible for unsuccessful aging [32].

Specifically, regarding the ArCSVD, the infiltration of immune cells such as macrophages, neutrophils, T cells, and NK cells causes inflammation, endothelial dysfunction and ischemia in the cerebral area [18].

In addition, the infiltration of senescent immune cells worsens the inflammatory state around the ischemic region through increased expression of inflammatory markers and alteration of the immune system. 

Moreover, infiltrating immune cells can produce ROS (reactive oxygen species), which cause oxidative stress on endothelial cells, leading to vascular remodeling and impaired vascular tone [33].

In the innate immune system, microglia is another important actor who plays an essential role in CNS phagocytosis, removing dead neurons, injured glial cells and debris of the myelin sheath. 

In aged people, the microglia also becomes senescent, and its dysfunction contributes to a low-grade inflammation state in CNS and neurodegenerative diseases [34,35].

Moreover, senescent microglia provokes impairment of phagocytosis and migration of immune cells, leading to senescent cells and debris accumulation. These cause chronic inflammation that damages cerebrovascular structures and neurons [35,36].

Ultimately, the alterations of the innate immune system responsible for the pathogenesis of ArCSVD are mainly due to the loss of the physiological synergy existing between immune and non-immune cells: chronic inflammation modifies the cellular microenvironment and triggers processes of cell degeneration that, together with the normal cellular aging, promote changes in the cerebral circulation.

Endothelial dysfunction, chronic inflammation promoted by senescent innate immune cells, oxidative stress, and impairment of microglial functions constitute the framework within which small vessel disease develops: it is a concatenation of molecular events that promotes the decline of the central nervous system and cognitive functions slowly and progressively.

The adaptive immune system also plays a crucial role in the immunological memory of the organism, which encourages the quick identification and elimination of specific pathogens by meeting specific sequences. 

**Figure 2:**
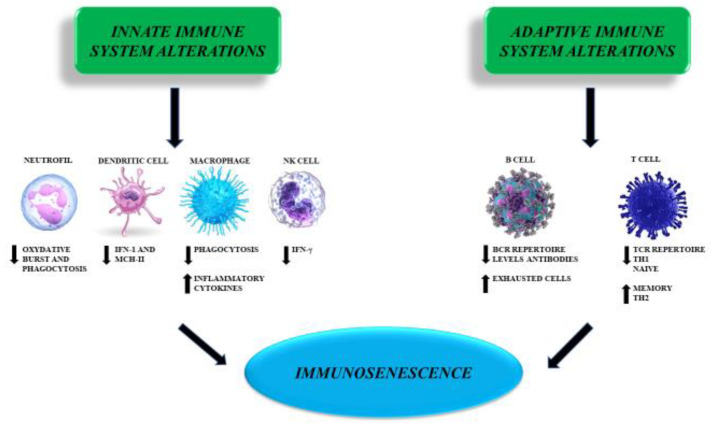
Alterations in the innate and adaptive immune system represent the foundation of those changes that the organism, as it ages, faces—immunosenescence and the alterations in molecular mechanisms that it presupposes are a cornerstone of the pathophysiology in ArCSVD.

With aging, two substantial changes occur: there is a reduction in naïve T cells resulting in a depletion of the TCR repertoire and an increase in memory T cells triggered by pathogens and aggressors previously met. In addition, naïve T-cell regeneration is limited because of thymic involution since puberty, continuous antigenic stimulations over the years, and reduced bone marrow supply for cellular production in the elderly [37] (Figure 2).

The above would explain the increased incidence of infections and cancer that characterizes aging and the decreased vaccination response [38].

However, these concepts have been challenged over the years: it is not entirely true that there is a depletion of the TCR heritage with aging, and it has been found that the replenishment of naïve T cells is partly obtained by the action of interleukin-7 and by the contribution of the Stem-Cell-like Memory T [39]. If, on the one hand, the aging of the adaptive immune system presupposes thymic involution, considering that the thymus is a metabolically “wasteful” organ, on the other hand, there is an attempt to allocate appropriate resources to protect the elderly. Furthermore, T cells fight internal and external antigens and can trigger the immune response against latent pathogens and reactivate under specific contexts, such as Cytomegalovirus (CMV) [40].

Although the available data are conflicting, CMV-driven recurrent antigenic stimulation is responsible for successful aging, maintenance of adaptive immune system functions, and vaccination response [41].

Concerning their features, aged T cells are divided into two classes: senescent and exhausted cells. Senescent cells are inert; exhausted cells are “dormant” and can be awakened by different stimulations by modulation of the surface receptors recovering their activity [42].

This aspect is essential because of its clinical relevance. For example, there are some cancers of the elderly, which is possible a modulation through the action at the level of checkpoint inhibitors (the surface receptors described above) [43].

Whatever stimulation activates the immune system, the molecular cascade of events that trigger the immune response is impaired in the elderly—alterations in immune synapse formation, mistakes in the process of constitution of transcription factors (mainly at the expense of those factors that are modulable). Among those described above, one of the most studied factors is a factor that regulates the assembly of molecules in cholesterol/ganglioside-containing nanoclusters [44].

Therefore, changes considered as part of aging in the past are the expression of an environmental role in the human body modifications and suggest that lifestyle and nutrition affect the process of immunosenescence [45] (Figure 2).

Many of the changes determined by the immunosenescence depend on the changes that characterize the whole organism and the cerebral district with aging: in aged spontaneously hypertensive rats (SHR), we observed an increased expression of adhesion molecules at the level of the cerebral microcirculation, then increased recruitment of lymphocytes in this district and dysfunction of small vessels resulting in thrombosis [46].

Moreover, antibodies against endothelial cells have been found to suggest the conditioning role of B lymphocytes in the genesis of endothelial dysfunction [47].

In the interdependent relationship between immunosenescence and inflammaging, it has also been observed a pathway of monocyte/macrophage activation able to promote the production of reactive oxygen species (ROS), to reduce the production of nitric oxide (NO), thus causing the increased expression of adhesion molecules, the hypertrophy of wall smooth muscle cells and the remodeling of the extracellular matrix by metalloproteinases [48], chronic inflammation and the differentiation of the role of immune cells in an atherogenic sense thus represent the keystone in the pathogenesis of ArCSVD. Consequently, they are considered the pathophysiological cornerstones of systemic and cerebral atherosclerosis specifically.

## 4. Age-Related Alterations in Non-Immune Cells: What Role Do They Play in ArCSVD?

In ArCSVD, old cerebral non-immune cells, such as endothelial cells, astrocytes, and oligodendrocytes, play an essential role in the destruction and dysfunction of the brain–blood barrier (BBB). Moreover, the immune cells can intercept these senescent cells and activate different responses. The endothelium is a critical system that plays a crucial role in the ArCSVD [49] through regulating vascular tone, vascular remodeling, and the balance of inflammation and coagulation. Many studies suggest that cellular oxidative stress and low-grade inflammation contribute to endothelial senescence [50].

The dysregulation of the vascular tone is one of the most critical factors of endothelial dysfunction. It is mainly due to reduced production of endothelial nitric oxide synthase (eNOS)-derived NO [51,52,53]: many factors such as hypertension, angiotensin II and aging can modify transcriptional and post-transcriptional eNOS signaling, leading to loss of function of eNOS and consequently reduction in NO serum levels [51,52,53].

The suppressed activity of eNOS causes modifications in vascular tone, reduced cerebral blood flow, increased oxidative stress and vulnerability to acute ischemia [51,52,53].

With aging of endothelial cells, therefore, a vicious circle is established through oxidative stress and inflammation, which progressively suppress the endothelial function and its ability to ensure a balance between vasodilating and vasoconstricting factors in favour of vasoconstriction, and this imbalance causes cerebrovascular dysfunction [54].

Aging causes activation of the Toll-like receptor Nuclear Factor kappa-B (TLR-NF-kB) molecular pathway, leading to increased production of inflammatory cytokines such as IL-1a, IL-1b, IL-6, TNF-a, and IFN-γ [55].

These inflammatory molecules and the NF-kB protein provoke the expression of nicotinamide adenine dinucleotide phosphate reduced (NADPH)-oxidase and, consequently, elevated levels of reactive oxygen species (ROS) [56].

In addition, the reactive oxygen species induce positive feedback on NF-kB activity and can recall circulating and residential immune cells [54], causing a dysregulate NO production by suppressing eNOS activity [57].

Vessel rarefaction is a common feature of ArCSVD, leading to a reduction in the tissue blood flow [58]. The imaging features of white matter hyperintensity or increased perivascular space and lacunar infarcts are the final consequences of cerebral vessel remodeling, leading to ischemic modifications in the regions vascularized by the responsible vessels.

Another factor involved in the pathogenesis of ArCSVD is dysfunction of the BBB due to the alterations of endothelial function and pericyte disorders. 

Immunosenescence induces those cellular changes described above, which, with other risk factors such as trauma or hypertension, provoke an increase in BBB leakage [59,60].

Although the pathophysiology of BBB dysfunction is complex, the most critical key factor of BBB leakage is endothelial dysfunction [61].

The chronic exposition of endothelial cells to the shear stress leads to modifications of the tight junctions and increased permeability of BBB [61].

As a result of BBB leakage, immune cells and circulating non-immune cells can infiltrate the brain tissue and provoke a low-grade inflammatory state [62]. With aging, brain damage is also caused by the transit of potentially harmful proteins across a deteriorated BBB [63].

In addition, the immune cells perpetuate the local inflammation and slow down the tissue reparation processes because of increased inflammatory cytokines and oxidative stress [64].

Aging causes an altered molecular microenvironment, which leads to an altered immune response and modification of the immune system. However, few studies have examined the possible relationships between BBB dysfunction and age-related modifications of peripheral cells of the innate or adaptive immune systems. Furthermore, many reports have suggested a possible contribution of the BBB changes in neuroimmune dysfunction. Some works have suggested that the early identification of pathological modifications of the BBB in living humans through imaging techniques such as PET, SPECT, and MRI could predict clinical symptoms such as cognitive impairment in aged patients [65].

Many studies suggest that modifications in BBB are one of the most critical factors contributing to the formation of white matter lesions and other secondary injuries in ArCSVD. These lesions found their radiological correspondents in the white matter hyperintensities (WMH) [65].

Furthermore, there is a connection between WMH and BBB leakage: the more significant the range of WMH, the more severe the BBB leakage [59].

In recent years, many studies have highlighted the role of Diffusion Tensor Imaging (DTI) MRI in CSVD [66,67]. DTI is a functional MRI technique aiming to detect changes in the neuronal network precociously. Williams et al., in a recent work, highlighted that the Automatic Diffusion Tensor Image Segmentation Technique (DSEG) is an accurate diagnostic tool able to assess brain microstructural damage in CSVD. Furthermore, DSEG seems to identify individuals who will develop dementia within five years. The authors, therefore, suggest that DSEG is a good technique to predict the risk of disease progression and development of dementia in subjects with risk factors for ArCSVD [16].

About the new frontiers of study in the field of the disease in question, in recent years, in vitro microfluidic BBB chips are becoming a kind of beacon and guidepost leading brain science research in the 21st century [68,69,70].

First of all, the multi-cell co-culture system provides potential for the investigation of CNS diseases. 

Because of the complex structure of the BBB, the conventional in vitro cell culture model fails to truly present the dynamic hemodynamics of BBB and the interaction between neurons. Therefore, miniaturized microfluidics-based BBB chips are commonly used to co-culture various cells on a small-sized chip to construct three-dimensional (3D) BBB or BBB-related organ disease models. By combining with other electrophysiological, biochemical sensors or equipment and imaging systems, it can, in real-time and quickly screen disease-related markers and evaluate drug efficacy [71].

BBB chips are non-invasive in vitro models capable of early identification of BBB leakage, considered one of the main risk factors for developing ArCSVD.

The detection of WMH on conventional MRI, the brain microstructural damage on DTI MRI and the presence of BBB leakage through in vitro BBB-Chips models could allow the early identification of patients who will develop ArCSVD, which is why early diagnosis and treatment of the underlying risk factors is crucial in blocking the development of the disease [72].

## 5. The Relationship between Immunosenescence and Inflammaging

Recent findings suggest that there is no one-way path in which immunosenescence produces inflammaging: indeed, there is a bidirectional pathway in which inflammaging induces and maintains immunosenescence and vice versa. This was understood by looking at what happens to T lymphocytes: in the elderly, there is an increase in memory of CD8+ T cells (in the past considered inert) characterized by the loss of naïve T cell surface markers, such as CD28 and CD27, and the appearance of new senescent markers such as KLRG1. Thus, the increase in the number of T memory cells, and consequently of B cells, with aging could be the expression of a chronic continuous antigenic stimulation analogous to the mechanism of inflammaging (for example, the trigger induced by CMV infection) [40].

The wide diffusion of CMV implies that the organism uses its immune energies to limit this specific infection in life. Thus, the T cell heritage is filled mainly by CMV-memory T cells. Previously, they were considered inactive and inert, but challenging data suggest that they are functionally active and contribute to inflammaging [73].

This chronic stimulation of the immune system causes the increase in the number of senescent T cells and the inflammageing; the consequence of chronic stimulation, however, is the appearance of exhausted cellular phenotypes with the remodeling of membrane receptors and the emergence of inhibitory receptors (PD-1, CTLA-4) [74].

Moreover, the B cell population is compromised and unable to fight against new harmful pathogens (altered clonal expansion, impaired antibody production), which is why there is a significant risk of developing infectious diseases and cancer [29].

This situation represents the dog biting its tail: the production of inflammatory mediators caused by inflammaging promotes cellular senescence and the decrease in the adaptive immune response; vice versa, the depletion of the adaptive immune mechanisms favours the stimulation of the innate immune system and the production of inflammatory mediators leading to inflammaging.

In this synoptic picture, it is possible to understand the physiopathological bases of the ArCSVD: inflammaging is recognized as an essential risk factor for vascular dementia and stroke by promoting the aging of the immune system and by acting synergistically with traditional risk factors (obesity, hypertension, diabetes mellitus, etc.) [75].

The progressive vascular damage of ArCSVD patients produces the release of central nervous system antigens in the peripheral circulation, generating the recruitment of lymphocytes in the brain tissue and the consequent dysfunctions [76].

In contrast, cerebral dysfunction is responsible for the progressive deterioration of the immune system, causing a continuous stimulation of the immune cells through the release of antigens and immunogenic molecules [77].

Pro-inflammatory molecules and activated immune cells are involved in the atherosclerotic process caused by inflammaging [78]. 

**Figure 3:**
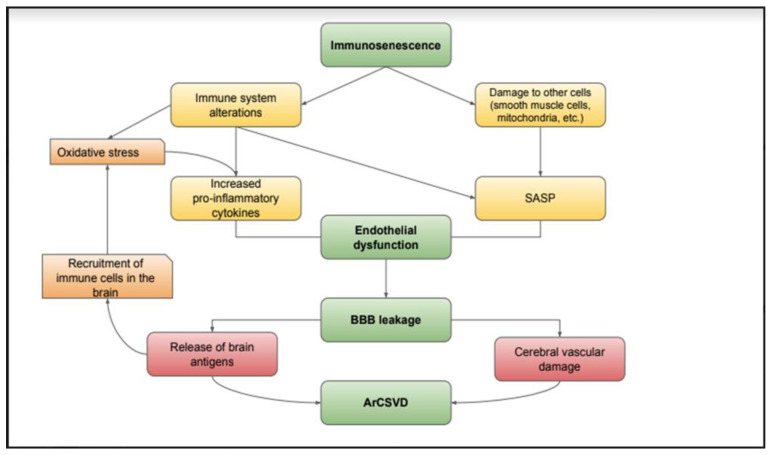
Aging causes alterations in the innate and adaptive immune system that lead to increased oxidative stress and endothelial dysfunction; this results in an alteration of the blood–brain barrier and a release of brain antigens into the systemic circulation, leading to a recall of immune cells in the brain. The amplification of the immune response feeds the inflammatory damage, establishing a self-feeding circuit that generates and worsens ArCSVD.

The correlation between immunosenescence and inflammaging is fundamental in understanding the pathogenesis of ArCSVD. With one-to-one correspondence, cellular aging and the loss of integrity of the blood–brain barrier favour the perpetual activation of the immune system, and the latter continuously worsens brain damage through a varied series of molecular alterations. Therefore, immunosenescence and inflammaging are two sides of the same coin and constitute a dynamic field of study and research to find therapeutic perspectives which interfere with these mechanisms [76,77,78] (Figure 3).

## 6. Immunotarget of Aging in Early Diagnosis and Therapeutic Perspectives of Cerebral Small Vessel Disease

At the brain level, it has been studied how the dysregulation of vascular homeostasis and the balance between the processes of vascular dilation and constriction, with the release of inflammatory cytokines, derive from endothelial dysfunction. These processes result from chronic inflammation, responsible at least partly for the onset of ischemic events. Endothelial dysfunction is a determinant key of vascular damage. Chronic inflammation induces the release of molecular mediators that trigger immunological reactions capable of stimulating and worsening brain damage, thus affecting the outcome of the disease [79].

Endothelial dysfunction is an early marker of cardiovascular disease: many studies showed the role of endothelial dysfunction and arterial stiffness as surrogate markers of vascular health.

Several works documented how, in diabetic patients suffering from microangiopathy, the indices of arterial stiffness (PWV and Aix) were higher than in the control group. In contrast, indices of endothelial function (RHI) and cognitive function (MMSE) were lower.

The authors speculated a possible correlation between increased arterial stiffness and decreased endothelial function, and mild cognitive impairment in this population [80].

Another scientific work, instead, evaluated the correlation between type 2 diabetes mellitus complicated by diabetic foot and the presence of white matter hyperintensity (WMH), also associated with alteration of omentin levels, and endothelial and cognitive performance indices [65,81].

In patients with diabetic foot, there was evidence of alteration of small vessels, as documented by the finding of hyperintensity of the white matter. Furthermore, the higher frequency of WMH lesions in these patients could be due to the higher degree of arterial stiffness, endothelial dysfunction and reduced serum levels of Omentin-1. Because of that, more robust cardiovascular prevention with angiotensin-converting enzyme inhibitors, angiotensin II receptor blockers, statins and aspirin, and the evaluation of the values of some adipokines could be necessary to identify the risk of brain-vessel dysfunction in the diabetic patient with organ complications [81].

It is essential to identify therapeutic targets and try to interfere with the mechanisms of immunosenescence. These therapeutic strategies could be specific or nonspecific. Concerning nonspecific therapies, it would be necessary to modify the risk factors that act by promoting chronic inflammation with consequent endothelial damage. Therefore, cardiovascular risk factors could be controlled (arterial hypertension, obesity, cigarette smoking, dyslipidemia, etc.), evaluating the possibility of introducing anti-platelet and anticoagulant therapy according to the individual patient’s ischaemic and haemorrhagic risk [82].

The use of specific categories of drugs could have a dual role, on the one hand, to allow the control of cardiovascular risk factors and, on the other hand, to limit inflammatory damage. Some studies have shown that therapy with statins, cilostazol, and ACE-i could stabilize endothelial cells and reduce the chronic inflammatory process [83].

Additionally, in some clinical trials, statins have significantly reduced the risk of cerebrovascular events in patients with hypercholesterolemia [84]. ACE-I-therapy has shown a more active role in preventing the progression of WMH in ArCSVD [85].

Shuzhen et al. proposed that lipoprotein-associated phospholipase A2 (Lp-PLA2) and superoxide dismutase (SOD) are independently related to cognitive dysfunction and injury. This means that the white matter hyperintensity (WMH) of ArCSVD could be considered a therapeutic target [86].

Considering the role of chronic inflammation and immunosenescence in the pathogenesis of ArCSVD, it would be interesting to consider using drugs already used for other neuroinflammatory diseases such as multiple sclerosis (fingolimod, natalizumab, dimethyl fumarate, and rituximab) in order to interfere with the action of the cytokines and inflammatory mediators involved [76].

About individual drugs, fingolimod is a drug commonly used in relapsing/remitting multiple sclerosis; it reduces circulating lymphocytes and, consequently, prevents them from escaping from the lymph nodes during a stroke. Therefore, this drug could contribute to the prevention of early infiltration of lymphocytes in the brain and could reduce thromboinflammation [87]. 

Fingolimod was used during the acute phase of ischemic stroke, and it showed an improvement in microvascular permeability and secondary damage [88].

Further studies are still needed to understand its precise role. In addition, a recent study found that fingolimod could induce the expression of VEGF in astrocytes. It, therefore, could stimulate the molecule S1PR3, which plays a role in the breakdown of the BBB and would be followed by the entry of pathogenic lymphocytes into the brain [89].

Natalizumab is another drug used in multiple sclerosis, and it has the function of blocking α4-integrin, which regulates the presence of lymphocytes (mainly T cells) in the central nervous system. The ACTION study considered administering natalizumab within 9 h of the onset of the acute ischemic event symptoms, but it did not find the effects of natalizumab on infarct growth. However, patients who received natalizumab had excellent cognitive outcomes at 90 days, especially in the small ischemic lesion group [90].

Dimethyl fumarate is also used in the treatment of multiple sclerosis. It has been documented that this drug is more effective and has fewer side effects than the other drugs tested. Above all, it has an essential role in the process of oxidative stress cells. This mechanism allows for the transcription of genes downstream of the activation of the antioxidant nuclear factor Nrf2 [91].

Molecules that mediate pericyte-EC interactions, such as TGF-β, and platelet-derived growth factor-BB (PDGF-BB), have been proposed as targets for the treatment of neurological disorders. Pericytes play a vital role in regulating various microvascular functions, such as angiogenesis, preservation of BBB, capillary blood flow, and migration of immune cells to the brain [92].

Some works have shown how pericytes can differentiate into neurons, microglia, and vascular cells after brain lesions in ischemic disease and hypoxia [93].

A trial in mice investigated how implantation of pericytes in the brain increased cerebral blood flow and could reduce pathological deposition of Aβ122 [94].

Thus, this evidence suggests that pericytes transplantation may be a promising approach for treating ArCSVD.

Inflammation is increasingly recognized as a risk factor for dementia, stroke, and ArCSVD [95].

As life expectancy continues to rise worldwide, the number of individuals living in the community with age-related diseases will increase, especially ArCSVD [95].

Inflammation not only acts through the aging of the immune system, but it also promotes the main cerebrovascular risk factors (such as obesity, hypertension, and type 2 diabetes) to trigger their damaging effects. For example, in ArCSVD patients, recurrent injuries such as mild stroke lead to BBB loss, central nervous system antigens release into the peripheral circulation, and infiltration of lymphocytes into brain tissue and related brain dysfunction. Brain dysfunction can further damage the immune system, forming a vicious cycle [77].

For this reason, the study of the role of the immune response during aging in the development of small vessel disease and the corresponding brain damage is of fundamental importance.

The role of immunosenescence in endothelial dysfunction and blood–brain barrier disorder has led to exciting aspects. Therefore, it could be a possible candidate for further study [96,97,98,99,100,101,102].

## 7. Conclusions

To fully understand the pathophysiological mechanisms and therefore to obtain effective therapeutic strategies against ArCSVD, it is necessary to have a holistic approach; it is necessary to counteract the known risk factors, and it is necessary to limit the effects of age-related alterations on the immune system. Moreover, through new therapeutic strategies, it is necessary to limit the effects of chronic age-related inflammation that represents an essential trigger for oxidative stress and endothelial dysfunction. 

The early diagnosis of ArCSVD, through serum markers of inflammation or functional or conventional MRI techniques, is crucial to treat early and strictly the risk factors that can cause disease progression and the onset of dementia.

Because the causative molecular mechanisms are not yet fully elucidated, the road of scientific research is stretched in this direction to discover as many aberrant processes and to ensure therapeutic weapons can improve the quality of life of people affected by them.

## Data Availability

Not applicable.

## References

[1] Mishra A., Chauhan G., Violleau M.H., Vojinovic D., Jian X., Bis J.C., Li S., Saba Y., Grenier-Boley B., Yang Q. (2019). Association of Variants in HTRA1 and NOTCH3 with MRI-Defined Extremes of Cerebral Small Vessel Disease in Older Subjects. Brain.

[2] Pantoni L. (2010). Cerebral Small Vessel Disease: From Pathogenesis and Clinical Characteristics to Therapeutic Challenges. Lancet Neurol..

[3] Khan U., Porteous L., Hassan A., Markus H.S. (2007). Risk Factor Profile of Cerebral Small Vessel Disease and Its Subtypes. J. Neurol. Neurosurg. Psychiatry.

[4] Huang Y., Yang C., Yuan R., Liu M., Hao Z. (2020). Association of Obstructive Sleep Apnea and Cerebral Small Vessel Disease: A Systematic Review and Meta-Analysis. Sleep.

[5] Han F., Zhai F.-F., Wang Q., Zhou L.-X., Ni J., Yao M., Li M.-L., Zhang S.-Y., Cui L.-Y., Jin Z.-Y. (2018). Prevalence and Risk Factors of Cerebral Small Vessel Disease in a Chinese Population-Based Sample. J. Stroke.

[6] Aw D., Silva A.B., Palmer D.B. (2007). Immunosenescence: Emerging Challenges for an Ageing Population. Immunology.

[7] Franceschi C., Bonafè M., Valensin S., Olivieri F., De Luca M., Ottaviani E., De Benedictis G. (2000). Inflamm-Aging. An Evolutionary Perspective on Immunosenescence. Ann. N. Y. Acad. Sci..

[8] Kennedy B.K., Berger S.L., Brunet A., Campisi J., Cuervo A.M., Epel E.S., Franceschi C., Lithgow G.J., Morimoto R.I., Pessin J.E. (2014). Geroscience: Linking Aging to Chronic Disease. Cell.

[9] Tieri P., Grignolio A., Zaikin A., Mishto M., Remondini D., Castellani G.C., Franceschi C. (2010). Network, Degeneracy and Bow Tie. Integrating Paradigms and Architectures to Grasp the Complexity of the Immune System. Theor. Biol. Med. Model..

[10] Franceschi C., Campisi J. (2014). Chronic Inflammation (Inflammaging) and Its Potential Contribution to Age-Associated Diseases. J. Gerontol. A. Biol. Sci. Med. Sci..

[11] Vitale G., Salvioli S., Franceschi C. (2013). Oxidative Stress and the Ageing Endocrine System. Nat. Rev. Endocrinol..

[12] Robbins P.D. (2017). Extracellular Vesicles and Aging. Stem Cell Investig..

[13] Franceschi C., Garagnani P., Vitale G., Capri M., Salvioli S. (2017). Inflammaging and “Garb-Aging”. Trends Endocrinol. Metab. TEM.

[14] Salminen A., Huuskonen J., Ojala J., Kauppinen A., Kaarniranta K., Suuronen T. (2008). Activation of Innate Immunity System during Aging: NF-KB Signaling Is the Molecular Culprit of Inflamm-Aging. Ageing Res. Rev..

[15] Schreiber S., Wilisch-Neumann A., Schreiber F., Assmann A., Scheumann V., Perosa V., Jandke S., Mawrin C., Carare R.O., Werring D.J. (2020). Invited Review: The Spectrum of Age-Related Small Vessel Diseases: Potential Overlap and Interactions of Amyloid and Nonamyloid Vasculopathies. Neuropathol. Appl. Neurobiol..

[16] Williams O.A., Zeestraten E.A., Benjamin P., Lambert C., Lawrence A.J., Mackinnon A.D.C., Morris R.G., Markus H.S., Barrick T.R., Charlton R.A. (2019). Predicting Dementia in Cerebral Small Vessel Disease Using an Automatic Diffusion Tensor Image Segmentation Technique. Stroke.

[17] Engelhardt B., Carare R.O., Bechmann I., Flügel A., Laman J.D., Weller R.O. (2016). Vascular, Glial, and Lymphatic Immune Gateways of the Central Nervous System. Acta Neuropathol..

[18] Kaiser D., Weise G., Möller K., Scheibe J., Pösel C., Baasch S., Gawlitza M. (2014). Spontaneous White Matter Damage, Cognitive Decline and Neuroinflammation in Middle-Aged Hypertensive Rats: An Animal Model of Early-Stage Cerebral Small Vessel Disease. Acta Neuropathol. Commun..

[19] Fibrinogen Inhibits Neurite Outgrowth via β3 Integrin-Mediated Phosphorylation of the EGF Receptor | PNAS. https://www.pnas.org/doi/10.1073/pnas.0704045104.

[20] Ryu J.K., Petersen M.A., Murray S.G., Baeten K.M., Meyer-Franke A., Chan J.P., Vagena E., Bedard C., Machado M.R., Rios Coronado P.E. (2015). Blood Coagulation Protein Fibrinogen Promotes Autoimmunity and Demyelination via Chemokine Release and Antigen Presentation. Nat. Commun..

[21] Frontiers | Registration. https://www.frontiersin.org/register.

[22] Avasarala J., Parti N. (2018). Can Aspirin Minimize Stroke Risk and New Lesion Formation in Multiple Sclerosis?. Front. Neurol..

[23] Cai W., Zhang K., Li P., Zhu L., Xu J., Yang B., Hu X., Lu Z., Chen J. (2017). Dysfunction of the Neurovascular Unit in Ischemic Stroke and Neurodegenerative Diseases: An Aging Effect. Ageing Res. Rev..

[24] Hu X., De Silva T.M., Chen J., Faraci F.M. (2017). Cerebral Vascular Disease and Neurovascular Injury in Ischemic Stroke. Circ. Res..

[25] Müller L., Fülöp T., Pawelec G. (2013). Immunosenescence in Vertebrates and Invertebrates. Immun. Ageing.

[26] Rivera A., Siracusa M.C., Yap G.S., Gause W.C. (2016). Innate Cell Communication Kick-Starts Pathogen-Specific Immunity. Nat. Immunol..

[27] Kleinnijenhuis J., Quintin J., Preijers F., Joosten L.A.B., Ifrim D.C., Saeed S., Jacobs C., van Loenhout J., de Jong D., Stunnenberg H.G. (2012). Bacille Calmette-Guerin Induces NOD2-Dependent Nonspecific Protection from Reinfection via Epigenetic Reprogramming of Monocytes. Proc. Natl. Acad. Sci. USA.

[28] Martínez de Toda I., Ceprián N., Díaz-Del Cerro E., De la Fuente M. (2021). The Role of Immune Cells in Oxi-Inflamm-Aging. Cells.

[29] Fülöp T., Dupuis G., Witkowski J.M., Larbi A. (2016). The Role of Immunosenescence in the Development of Age-Related Diseases. Rev. Investig. Clin. Organo Hosp. Enfermedades Nutr..

[30] Fulop T., Dupuis G., Baehl S., Le Page A., Bourgade K., Frost E., Witkowski J.M., Pawelec G., Larbi A., Cunnane S. (2016). From Inflamm-Aging to Immune-Paralysis: A Slippery Slope during Aging for Immune-Adaptation. Biogerontology.

[31] Frontiers | Macrophage Metabolism As Therapeutic Target for Cancer, Atherosclerosis, and Obesity | Immunology. https://www.frontiersin.org/articles/10.3389/fimmu.2017.00289/full.

[32] Rose G., Santoro A., Salvioli S. (2017). Mitochondria and Mitochondria-Induced Signalling Molecules as Longevity Determinants. Mech. Ageing Dev..

[33] Frontiers | Cerebral Small Vessel Disease: Targeting Oxidative Stress as a Novel Therapeutic Strategy? | Pharmacology. https://www.frontiersin.org/articles/10.3389/fphar.2016.00061/full.

[34] Niraula A., Sheridan J.F., Godbout J.P. (2017). Microglia Priming with Aging and Stress. Neuropsychopharmacol. Off. Publ. Am. Coll. Neuropsychopharmacol..

[35] Plaza-Zabala A., Sierra-Torre V., Sierra A. (2017). Autophagy and Microglia: Novel Partners in Neurodegeneration and Aging. Int. J. Mol. Sci..

[36] Harry G.J. (2013). Microglia during Development and Aging. Pharmacol. Ther..

[37] Goronzy J.J., Fang F., Cavanagh M.M., Qi Q., Weyand C.M. (2015). Naive T Cell Maintenance and Function in Human Aging. J. Immunol. Baltim. Md 1950.

[38] Pawelec G. (2017). Immunosenescence and Cancer. Biogerontology.

[39] Gattinoni L., Speiser D.E., Lichterfeld M., Bonini C. (2017). T Memory Stem Cells in Health and Disease. Nat. Med..

[40] Pawelec G. (2014). Immunosenenescence: Role of Cytomegalovirus. Exp. Gerontol..

[41] McElhaney J.E., Garneau H., Camous X., Dupuis G., Pawelec G., Baehl S., Tessier D., Frost E.H., Frasca D., Larbi A. (2015). Predictors of the Antibody Response to Influenza Vaccination in Older Adults with Type 2 Diabetes. BMJ Open Diabetes Res. Care.

[42] Zou W., Wolchok J.D., Chen L. (2016). PD-L1 (B7-H1) and PD-1 Pathway Blockade for Cancer Therapy: Mechanisms, Response Biomarkers, and Combinations. Sci. Transl. Med..

[43] Elias R., Karantanos T., Sira E., Hartshorn K.L. (2017). Immunotherapy Comes of Age: Immune Aging & Checkpoint Inhibitors. J. Geriatr. Oncol..

[44] Fulop T., Le Page A., Garneau H., Azimi N., Baehl S., Dupuis G., Pawelec G., Larbi A. (2012). Aging, Immunosenescence and Membrane Rafts: The Lipid Connection. Longev. Heal..

[45] Turner J.E., Brum P.C. (2017). Does Regular Exercise Counter T Cell Immunosenescence Reducing the Risk of Developing Cancer and Promoting Successful Treatment of Malignancies?. Oxid. Med. Cell. Longev..

[46] Role of T Lymphocytes in Angiotensin II–Mediated Microvascular Thrombosis | Hypertension. https://www.ahajournals.org/doi/10.1161/hypertensionaha.111.173856.

[47] Kimura A., Sakurai T., Yamada M., Koumura A., Hayashi Y., Tanaka Y., Hozumi I., Ohtaki H., Chousa M., Takemura M. (2012). Anti-Endothelial Cell Antibodies in Patients with Cerebral Small Vessel Disease. Curr. Neurovasc. Res..

[48] Virdis A., Neves M.F., Amiri F., Touyz R.M., Schiffrin E.L. (2004). Role of NAD(P)H Oxidase on Vascular Alterations in Angiotensin II-Infused Mice. J. Hypertens..

[49] Poggesi A., Pasi M., Pescini F., Pantoni L., Inzitari D. (2016). Circulating Biologic Markers of Endothelial Dysfunction in Cerebral Small Vessel Disease: A Review. J. Cereb. Blood Flow Metab. Off. J. Int. Soc. Cereb. Blood Flow Metab..

[50] Buford T.W. (2016). Hypertension and Aging. Ageing Res. Rev..

[51] McCarty M.F. (2015). NADPH Oxidase Activity in Cerebral Arterioles Is a Key Mediator of Cerebral Small Vessel Disease-Implications for Prevention. Healthc. Basel Switz..

[52] Berkowitz D.E., White R., Li D., Minhas K.M., Cernetich A., Kim S., Burke S., Shoukas A.A., Nyhan D., Champion H.C. (2003). Arginase Reciprocally Regulates Nitric Oxide Synthase Activity and Contributes to Endothelial Dysfunction in Aging Blood Vessels. Circulation.

[53] Vanhoutte P.M., Zhao Y., Xu A., Leung S.W.S. (2016). Thirty Years of Saying NO: Sources, Fate, Actions, and Misfortunes of the Endothelium-Derived Vasodilator Mediator. Circ. Res..

[54] Donato A.J., Morgan R.G., Walker A.E., Lesniewski L.A. (2015). Cellular and Molecular Biology of Aging Endothelial Cells. J. Mol. Cell. Cardiol..

[55] Mun G.I., Boo Y.C. (2010). Identification of CD44 as a Senescence-Induced Cell Adhesion Gene Responsible for the Enhanced Monocyte Recruitment to Senescent Endothelial Cells. Am. J. Physiol. Heart Circ. Physiol..

[56] Pierce G.L., Lesniewski L.A., Lawson B.R., Beske S.D., Seals D.R. (2009). Nuclear Factor-{kappa}B Activation Contributes to Vascular Endothelial Dysfunction via Oxidative Stress in Overweight/Obese Middle-Aged and Older Humans. Circulation.

[57] Lubos E., Handy D.E., Loscalzo J. (2008). Role of Oxidative Stress and Nitric Oxide in Atherothrombosis. Front. Biosci. J. Virtual Libr..

[58] Liu Y., Dong Y.-H., Lyu P.-Y., Chen W.-H., Li R. (2018). Hypertension-Induced Cerebral Small Vessel Disease Leading to Cognitive Impairment. Chin. Med. J..

[59] Zhang C.E., Wong S.M., van de Haar H.J., Staals J., Jansen J.F.A., Jeukens C.R.L.P.N., Hofman P.A.M., van Oostenbrugge R.J., Backes W.H. (2017). Blood-Brain Barrier Leakage Is More Widespread in Patients with Cerebral Small Vessel Disease. Neurology.

[60] Muñoz Maniega S., Chappell F.M., Valdés Hernández M.C., Armitage P.A., Makin S.D., Heye A.K., Thrippleton M.J., Sakka E., Shuler K., Dennis M.S. (2017). Integrity of Normal-Appearing White Matter: Influence of Age, Visible Lesion Burden and Hypertension in Patients with Small-Vessel Disease. J. Cereb. Blood Flow Metab. Off. J. Int. Soc. Cereb. Blood Flow Metab..

[61] Wardlaw J.M., Sandercock P.A.G., Dennis M.S., Starr J. (2003). Is Breakdown of the Blood-Brain Barrier Responsible for Lacunar Stroke, Leukoaraiosis, and Dementia?. Stroke.

[62] Henning E.C., Warach S., Spatz M. (2010). Hypertension-Induced Vascular Remodeling Contributes to Reduced Cerebral Perfusion and the Development of Spontaneous Stroke in Aged SHRSP Rats. J. Cereb. Blood Flow Metab. Off. J. Int. Soc. Cereb. Blood Flow Metab..

[63] Montagne A., Barnes S.R., Sweeney M.D., Halliday M.R., Sagare A.P., Zhao Z., Toga A.W., Jacobs R.E., Liu C.Y., Amezcua L. (2015). Blood-Brain Barrier Breakdown in the Aging Human Hippocampus. Neuron.

[64] Obermeier B., Daneman R., Ransohoff R.M. (2013). Development, Maintenance and Disruption of the Blood-Brain Barrier. Nat. Med..

[65] Joutel A., Chabriat H. (2017). Pathogenesis of White Matter Changes in Cerebral Small Vessel Diseases: Beyond Vessel-Intrinsic Mechanisms. Clin. Sci. Lond. Engl. 1979.

[66] Dykan I.M., Golovchenko Y.I., Loganovsky K.M., Semonova O.V., Myronyak L.A., Babkina T.M., Kuts K.V., Kobzar I.O., Gresko M.V., Loganovska T.K. (2020). Diffusion tensor magnetic resonance imaging in early diagnosis of structural changes in brain white matter in small vessel disease associated with arterial hypertension and ionizing radiation. Probl. Radiatsiinoi Medytsyny Ta Radiobiolohii.

[67] Xie Y., Xie L., Kang F., Jiang J., Yao T., Li Y., Mao G., Wu D. (2021). Association between Diffusion Tensor Imaging Findings and Domain-Specific Cognitive Impairment in Cerebral Small Vessel Disease: A Protocol for Systematic Review and Meta-Analysis. BMJ Open.

[68] Esch E.W., Bahinski A., Huh D. (2015). Organs-on-Chips at the Frontiers of Drug Discovery. Nat. Rev. Drug Discov..

[69] Peck R.W., Hinojosa C.D., Hamilton G.A. (2020). Organs-on-Chips in Clinical Pharmacology: Putting the Patient Into the Center of Treatment Selection and Drug Development. Clin. Pharmacol. Ther..

[70] Rothbauer M., Rosser J.M., Zirath H., Ertl P. (2019). Tomorrow Today: Organ-on-a-Chip Advances towards Clinically Relevant Pharmaceutical and Medical in Vitro Models. Curr. Opin. Biotechnol..

[71] Wang X., Hou Y., Ai X., Sun J., Xu B., Meng X., Zhang Y., Zhang S. (2020). Potential Applications of Microfluidics Based Blood Brain Barrier (BBB)-on-Chips for in Vitro Drug Development. Biomed. Pharmacother. Biomedecine Pharmacother..

[72] Holm J.E., Bury L., Suda K.T. (1996). The Relationship between Stress, Headache, and the Menstrual Cycle in Young Female Migraineurs. Headache.

[73] Akbar A.N., Henson S.M., Lanna A. (2016). Senescence of T Lymphocytes: Implications for Enhancing Human Immunity. Trends Immunol..

[74] Wherry E.J., Kurachi M. (2015). Molecular and Cellular Insights into T cell Exhaustion. Nat. Rev. Immunol..

[75] Low A., Mak E., Rowe J.B., Markus H.S., O’Brien J.T. (2019). Inflammation and Cerebral Small Vessel Disease: A Systematic Review. Ageing Res. Rev..

[76] Fu Y., Yan Y. (2018). Emerging Role of Immunity in Cerebral Small Vessel Disease. Front. Immunol..

[77] Calder P.C., Bosco N., Bourdet-Sicard R., Capuron L., Delzenne N., Doré J., Franceschi C., Lehtinen M.J., Recker T., Salvioli S. (2017). Health Relevance of the Modification of Low Grade Inflammation in Ageing (Inflammageing) and the Role of Nutrition. Ageing Res. Rev..

[78] Liberale L., Diaz-Cañestro C., Bonetti N.R., Paneni F., Akhmedov A., Beer J.H., Montecucco F., Lüscher T.F., Camici G.G. (2018). Post-Ischaemic Administration of the Murine Canakinumab-Surrogate Antibody Improves Outcome in Experimental Stroke. Eur. Heart J..

[79] Tuttolomondo A., Daidone M., Pinto A. (2020). Endothelial Dysfunction and Inflammation in Ischemic Stroke Pathogenesis. Curr. Pharm. Des..

[80] Tuttolomondo A., Casuccio A., Guercio G., Maida C., Del Cuore A., Di Raimondo D., Simonetta I., Di Bona D., Pecoraro R., Della Corte V. (2017). Arterial Stiffness, Endothelial and Cognitive Function in Subjects with Type 2 Diabetes in Accordance with Absence or Presence of Diabetic Foot Syndrome. Cardiovasc. Diabetol..

[81] Tuttolomondo A., Di Raimondo D., Casuccio A., Guercio G., Del Cuore A., Puleo M.G., Della Corte V., Bellia C., Caronia A., Maida C. (2019). Endothelial Function, Adipokine Serum Levels and White Matter Hyperintesities in Subjects with Diabetic Foot Syndrome. J. Clin. Endocrinol. Metab..

[82] Bath P.M., Wardlaw J.M. (2015). Pharmacological Treatment and Prevention of Cerebral Small Vessel Disease: A Review of Potential Interventions. Int. J. Stroke Off. J. Int. Stroke Soc..

[83] Rajani R.M., Quick S., Ruigrok S.R., Graham D., Harris S.E., Verhaaren B.F.J., Fornage M., Seshadri S., Atanur S.S., Dominiczak A.F. (2018). Reversal of Endothelial Dysfunction Reduces White Matter Vulnerability in Cerebral Small Vessel Disease in Rats. Sci. Transl. Med..

[84] Fu J.H., Mok V., Lam W., Wong A., Chu W., Xiong Y., Ng P.W., Tsoi T.H., Yeung V., Wong K.S. (2010). Effects of Statins on Progression of Subclinical Brain Infarct. Cerebrovasc. Dis. Basel Switz..

[85] Dufouil C., Chalmers J., Coskun O., Besançon V., Bousser M.-G., Guillon P., MacMahon S., Mazoyer B., Neal B., Woodward M. (2005). Effects of Blood Pressure Lowering on Cerebral White Matter Hyperintensities in Patients with Stroke: The PROGRESS (Perindopril Protection Against Recurrent Stroke Study) Magnetic Resonance Imaging Substudy. Circulation.

[86] Zhu S., Wei X., Yang X., Huang Z., Chang Z., Xie F., Yang Q., Ding C., Xiang W., Yang H. (2019). Plasma Lipoprotein-Associated Phospholipase A2 and Superoxide Dismutase are Independent Predicators of Cognitive Impairment in Cerebral Small Vessel Disease Patients: Diagnosis and Assessment. Aging Dis..

[87] Liesz A., Sun L., Zhou W., Schwarting S., Mracsko E., Zorn M., Bauer H., Sommer C., Veltkamp R. (2011). FTY720 Reduces Post-Ischemic Brain Lymphocyte Influx but Does Not Improve Outcome in Permanent Murine Cerebral Ischemia. PLoS ONE.

[88] Fu Y., Hao J., Zhang N., Ren L., Sun N., Li Y.-J., Yan Y., Huang D., Yu C., Shi F.-D. (2014). Fingolimod for the Treatment of Intracerebral Hemorrhage: A 2-Arm Proof-of-Concept Study. JAMA Neurol..

[89] Dusaban S.S., Chun J., Rosen H., Purcell N.H., Brown J.H. (2017). Sphingosine 1-Phosphate Receptor 3 and RhoA Signaling Mediate Inflammatory Gene Expression in Astrocytes. J. Neuroinflammation.

[90] Elkins J., Veltkamp R., Montaner J., Johnston S.C., Singhal A.B., Becker K., Lansberg M.G., Tang W., Chang I., Muralidharan K. (2017). Safety and Efficacy of Natalizumab in Patients with Acute Ischaemic Stroke (ACTION): A Randomised, Placebo-Controlled, Double-Blind Phase 2 Trial. Lancet Neurol..

[91] Linker R.A., Lee D.-H., Ryan S., van Dam A.M., Conrad R., Bista P., Zeng W., Hronowsky X., Buko A., Chollate S. (2011). Fumaric Acid Esters Exert Neuroprotective Effects in Neuroinflammation via Activation of the Nrf2 Antioxidant Pathway. Brain J. Neurol..

[92] Cheng J., Korte N., Nortley R., Sethi H., Tang Y., Attwell D. (2018). Targeting Pericytes for Therapeutic Approaches to Neurological Disorders. Acta Neuropathol..

[93] Human Brain Vascular Pericytes (HBVP) | Creative Bioarray. https://www.creative-bioarray.com/Human-Brain-Vascular-Pericytes-HBVP-CSC-7825W-item-1880.htm?gclid=EAIaIQobChMIrpa7qs7B9wIVeYxoCR3bZQcJEAAYASAAEgKJsfD_BwE.

[94] Tachibana M., Yamazaki Y., Liu C.-C., Bu G., Kanekiyo T. (2018). Pericyte Implantation in the Brain Enhances Cerebral Blood Flow and Reduces Amyloid-β Pathology in Amyloid Model Mice. Exp. Neurol..

[95] Hilal S., Mok V., Youn Y.C., Wong A., Ikram M.K., Chen C.L.-H. (2017). Prevalence, Risk Factors and Consequences of Cerebral Small Vessel Diseases: Data from Three Asian Countries. J. Neurol. Neurosurg. Psychiatry.

[96] Wardlaw J.M., Smith C., Dichgans M. (2013). Mechanisms of Sporadic Cerebral Small Vessel Disease: Insights from Neuroimaging. Lancet Neurol..

[97] Pinto A., Di Raimondo D., Tuttolomondo A., Fernandez P., Arnao V., Licata G. (2006). Twenty-four hour ambulatory blood pressure monitoring to evaluate effects on blood pressure of physical activity in hypertensive patients. Clin. J. Sport Med..

[98] Basili S., Raparelli V., Napoleone L., Talerico G., Corazza G.R., Perticone F., Sacerdoti D., Andriulli A., Licata A., Pietrangelo A. (2018). Platelet Count Does Not Predict Bleeding in Cirrhotic Patients: Results from the PRO-LIVER Study. Am. J. Gastroenterol..

[99] Siragusa S., Malato A., Saccullo G., Iorio A., Di Ianni M., Caracciolo C., Coco L.L., Raso S., Santoro M., Guarneri F.P. (2011). Residual vein thrombosis for assessing duration of anticoagulation after unprovoked deep vein thrombosis of the lower limbs: The extended DACUS study. Am. J. Hematol..

[100] Zanoli L., Boutouyrie P., Fatuzzo P., Granata A., Lentini P., Oztürk K., Cappello M., Theocharidou E., Tuttolomondo A., Pinto A. (2017). Inflammation and Aortic Stiffness: An Individual Participant Data Meta-Analysis in Patients With Inflammatory Bowel Disease. J. Am. Heart Assoc..

[101] Zanoli L., Ozturk K., Cappello M., Inserra G., Geraci G., Tuttolomondo A., Torres D., Pinto A., Duminuco A., Riguccio G. (2019). Inflammation and Aortic Pulse Wave Velocity: A Multicenter Longitudinal Study in Patients With Inflammatory Bowel Disease. J. Am. Heart Assoc..

[102] Maida C.D., Norrito R.L., Daidone M., Tuttolomondo A., Pinto A. (2020). Neuroinflammatory Mechanisms in Ischemic Stroke: Focus on Cardioembolic Stroke, Background, and Therapeutic Approaches. Int. J. Mol. Sci..

